# The Diagnosis of Malignant Diseases of the Mouth*Read before the Susquehanna Dental Association of Pennsylvania, at its annual meeting, Delaware Water Gap, May 26, 1914, and reprinted from the Dental Cosmos for Nov. 1914.

**Published:** 1914-12-15

**Authors:** W. J. Roe

**Affiliations:** Philadelphia, Pa.


### THE DIAGNOSIS OF MALIGNANT DISEASES OF THE MOUTH.*

BY W. J. ROE, M.D., D.D.S., PHILADELPHIA, PA.

Malignant is a term applied to diseases which increase in intensity with rapidity or which proceed to a fatal end. There are, however, varying degrees of malignancy. The terms *low, average,* and *high* malignancy are used to designate the degree of rapidity of increase in intensity. Although the majority of cases correspond to the average type, cases of both high and low malignancy and occasionally of an extreme type are not infrequently seen. All proceed to a fatal termination. Only two diseases of the mouth in medicine and surgery answer to the term "malignant," these being carcinoma and sarcoma, the malignant neoplasms or tumors, the diagnosis or recognition of which is dependent upon the operator's familiarity with their clinical and pathological phenomena. The conception clinically of a disease increasing in intensity with any degree of rapidity to a fatal end will establish the diagnosis of malignancy, and the application of the pathological anatomy in the same will clinch the differential diagnosis between carcinoma or sarcoma.

There are other diseases of the mouth that in a percentage of cases become malignant in type, but not invariably so, and they are, therefore, not malignant diseases. Among these are cancrum oris, carbuncle, glanders or farcy, malignant pustule or anthrax, diptheria, cases of virulent and rapidly spreading infections, actinomycosis, syphilis, tuberculosis, tetanus, rabies, and scorbutus or scurvy.

*Read before the Susquehanna Dental Association of Pennsylvania, at its annual meeting, Delaware Water Gap, May 26, 1914, and reprinted from the *Dental Cosmos* for Nov. 1914.

CARCINOMA AND SARCOMA.

Although the histological diagnosis is the only accepted one in surgery for statistics, owing to the many mistakes that would otherwise occur, it is not always dependable. The microscopist may through delay or faulty handling of the specimen, misplacement, or improper selection of the section to be cut, or insufficient cuts or mounts, be led into error unavoidably, or through haste or many other circumstances be faulty in his conclusions. I have heard a well-known pathologist, now professor of that department in one of our southern universities, say that he would just as soon accept the clinical diagnosis of Dr. W. Joseph Hearn of Philadelphia as he would his findings under the microscope. It is not often convenient or even desirable to obtain a portion of a tumor for histological diagnosis, for many reasons, the most important of which I believe to be the possible and in many cases probable danger of producing regional and metastatic dissemination as a result of the surgical traumatism and consequent inflammation. Neither is the blood examination of definite value, as the toxins or isohemolysis of the serum of the blood in cases of carcinoma and sarcoma have a similar hemolytic action to that in cases of tuberculosis and syphilis. The presence of isohemolysis, together with the marked and progressive anemia characterized by a deficient number of erythrocytes and reduced hemoglobin, with leucocytes about normal, would point strongly to one of the three conditions. The progressive anemia is not influenced by medical treatment, as it usually is in cases of tuberculosis and syphilis

The Roentgen ray is of distinct value and a very reliable aid in establishing a diagnosis, as both sarcoma and carcinoma destroy bone. Carcinoma reaching the maxillary bones by extension from a primary site in the epithelium of the skin or mucous membrane, or its extension along ducts and their tributaries in the associated glands, involves bone secondarily. Sarcoma may begin primarily within the bone or its periosteum and involve bone from its early development or by extension from a primary site external to the periosteum but necessarily beneath the skin and mucous membrane.

The loss or disappearance of bone tissue in the absence of suppuration of bone, producing molecular disintegration or necrosis; previous loss from surgical traumatism, injury, or disease; cystic expansion and rarefaction, tuberculous or syphilitic granulomata, when shown in the skiagraph with fairly well-defined limitations, are distinctly diagnostic. When the regional loss of bone substance is replaced with tissue of greatly lessened density to the palpating finger in contrast to the normal surface resistance, smoothness, and outline of adjacent bone, together with altered and usually increased contour, the diagnosis becomes more certain.

### COMPLICATION WITH CYST.

It is not always a very easy task to exclude the previously mentioned conditions, and of even more importance to be considered is the fact that there may be associated with the sarcoma or carcinoma or occurring as a complication or incident, any one or several of these. The presence of a dental or dentigerous cyst would not exclude the development of sarcoma or carcinoma, although it would be comparatively infrequent in smaller cysts, but relatively more frequent in larger cysts of long standing. If in a large dental or dentigerous cyst a local area shows marked and quite rapid thickening, this is strongly suggestive of a malignant development beginning in the cyst wall. In four such cases I was able to make a diagnosis from the rather abrupt and local development, together with the regional loss of bone tissue in contrast to the rarefied and more definite outline of bone in the cyst wall and its region.

In dental and dentigerous cysts, the slow and uneventful development, except for the increased contour and often the displacement of teeth, the parchment-like crackling of the thin outer wall, fluctuation, the rarefaction of the bone, and the clear outline as shown by the skiagraph—and less distinctly by transillumination—are diagnostic. If yet in doubt, it is an easy task to introduce an aspirating needle through the thinnest portion of the wall. The finding of misplaced teeth would not prove the presence of a dentigerous cyst, as they might be unerupted malposed teeth incidental to a case of malignant disease. In virulent and rapidly spreading infections of bone tissue, the bone will be destroyed *en masse* and the evidence of sequestra be present, or in ordinary suppuration of bone and abscess formation there is the presence of a purulent cavity in bone with its sinus.

It is always well to ascertain if any previous operation or injury had occurred, and especially if any evidence of previous scar tissue is present, so as to correctly interpret the skiagram.

The skiagram will often reveal the cause of obscure enlargements incidentally discovered by the patient, dentist, or physician, of any portion of the maxillary bones, due to unerupted, malposed or impacted teeth, that would otherwise be considered a probable sarcoma.

### FACTORS IN DIFFERENTIAL DIAGNOSIS.

To make a differential diagnosis between carcinoma and sarcoma, the patient's age and the tissue primarily involved are of the greatest importance. Carcinoma of the mouth is quite rare before thirty years of age, but becomes more frequent in each decade of life subsequently. Sarcoma is more frequent before the thirtieth year, and decreases in each subsequent decade. If the primary site is the epithelium of the mouth, the case would be one of carcinoma, if the connective tissue, sarcoma. It is not always possible to obtain this knowledge, except when the case is seen early, or the patient is sufficiently intelligent and accurate in observation. This is impossible in a primary cacinoma of the epithelium of the maxillary sinus, and the diagnosis cannot be made in patients in the cancerous age, except for the fact that the frequency of sarcoma of the maxilla involving the mucous membrane of the sinus and occupying the same is much greater. However, in carcinoma of the mucous membrane of the maxillary sinus there are frequently more associated symptoms which occur from the surface of exposed carcinomatous tissue, such as sanguinous sero-mucous or muco-purulent discharge into the nasal cavity, which from decomposition is often quite offensive.

### DIFFERENTIATION FROM EMPYEMA OF THE ANTRUM.

I also want to call attention to the fact that the tissues of carcinoma and sarcoma can become invaded and infected with pathogenic germs as in normal tissue—suppuration, abscess, or necrosis taking place owing to the invasion of pyogenic micro-organisms. When suppuration occurs in carcinomatous or sarcomatous tissue, partly or completely filling the maxillary sinus, a portion of the tissue is destroyed and an abscess results. If the patient has not been under observation before such a complication develops, and is unable to give a clear history of preceding trouble, it is often very difficult to make a diagnosis other than empyema of the maxillary sinus. In empyema of the maxillary sinus the surrounding bone is practically never necrosed or destroyed, and retains its regular contour, surface smoothness, and resistance, except in rare cases when the pus is retained under considerable pressure, and the bony walls are somewhat displaced or bulged.

In malignant diseases complicated with suppuration and abscess, the carcinoma or sarcoma has probably destroyed portions of the wall and extended into surrounding tissue, which gives irregular density without the normal resistance of the bone. In malignant diseases the lost tissue is soon replaced, providing the suppuration is not so active that the tissue is destroyed as rapidly as it is formed. Even though the abscess in the region of the maxillary sinus did continue, the sarcoma or carcinoma would extend in the periphery, and develop more rapidly owing to the inflammatory stimulus. In empyema the improvement would be rapid and permanent in response to treatment.

Here also the skiagraph will show the loss of bone, but transillumination will not afford as reliable evidence. If there is reasonable doubt, and the treatment for empyema of the sinus has been decided upon, it would be best to secure, if possible, a section of tissue from the probable site of the malignant tissue and have it examined histologically. Scrapings from the maxillary sinus would not prove a reliable specimen.

DESTRUCTION OF THE ALVEOLUS. A CAUTION IN EXTRACTING.

Another important evidence of destruction of bone by sarcoma or carcinoma when it involves the alveolar process is the gradual loss of bony support to the teeth, which become functionless and are often detached. When the bone is partially destroyed, the remaining portions will frequently come away with the tooth in extracting, and portions of the malignant tissue also will often come away.

Dentists are frequently charged by ignorant patients with having caused the malignant disease when they extracted a troublesome and loosened tooth from an area of carcinoma and sarcoma which had already destroyed the alveolus, and was the cause of the suffering. rather than the tooth. To have allowed the tooth to remain would have prevented the criticism, and also the possibility of increasing the danger of regional or general dissemination from the surgical traumatism.

TUBERCULOUS LESIONS OF THE MOUTH.

Tuberculosis involving the tissues of the mouth can begin in the surface or deeper structures, and may be mistaken for carcinoma or sarcoma. Tuberculous lesions of the mouth that persist and become severe are not common, and are invariably secondary infections from pulmonary tuberculosis of an advanced stage, and should be strongly suspected upon finding the pulmonary involvement. It must be remembered that patients suffering from advanced pulmonary tuberculosis may develop carcinoma or sarcoma, and that the surface of a malignant tumor in the mouth in a phthisical patient would be invariably contaminated with tubercle bacilli, so that their presence would not prove the absence of malignant disease. Closer observation for a short period would show in sarcoma or carcinoma a steady increase in the contour and size of the tumor, whereas in tuberculosis, although the disease would have probably extended, there would be further loss of contour by degeneration and ulceration, or it might have been arrested by treatment.

A tubercle developing in the deeper tissues of the mouth will usually progress to a tuberculous or so-called cold abscess, which in its early stages is difficult to diagnose from sarcoma, especially if it is a primary tubercle. Tuberculous involvement of the maxillary bones secondary to tuberculous cervical adentitis does occur, and should be suspected. Small tuberculous abscesses not infrequently are found in the soft tissues adjacent to lesions of the teeth or gums which respond readily to treatment.

### SYPHILITIC LESIONS.

Syphilitic lesions of the mouth, such as the primary ulcer or chancre and the tertiary granulomata or gummata, are more quickly and positively diagnosed by means of the Wassermann test. But in the absence of that evidence in the initial lesion, we find that the abrupt, rapid development of the ulcer with indurated base and the almost synchronous and marked regional enlargement of the lymphatic glands stand in marked contrast to the more slowly developing epithelioma, with less induration and late involvement of the regional glands; also the chancre is more frequent before the cancerous age than after. Again I would emphasize the frequency of cancer developing in a patient with also a positive Wassermann reaction. Here the persistent development uninfluenced by treatment, or a histological examination, will establish the diagnosis.

### ACTINOMYCOSIS.

Actinomycosis is comparatively infrequent in man, but when present it is found most frequently in the region of the mouth and involving the maxillary bones, usually the mandible. The irregular ridge-like development and sinus formation, with the escaping characteristic yellow gritty granules contained in the pus—which under the microscope prove to be actinomyces, or ray fungus—make the diagnosis.

### ODONTOMA.

Odontoma, another comparatively rare disease, can be definitely diagnosed by means of the roentgen rays, yet without them its slow development and solidity, definition of location, contour, and outline would be distinctive, and an exploratory incision or puncture would establish a diagnosis. Osteoma is so rare in the maxillary bones that it is scarcely to be considered in the diagnosis of carcinoma or sarcoma.

### DERMOID CYSTS.

Dermoid cysts developing in the mandible or maxilla are occasionally confused with sarcoma, but are so similar to dental and dentigerous cysts in expansion and rarefaction of bone and displacement of teeth that the same methods of diagnosis are available. Dermoids of the floor of the mouth or soft palate, and retention cysts, such as mucous cysts of the lips and ranula of the floor of the mouth, are recognized by the fluctuation, elasticity, and translucence, or doughy resistance.

### SUMMARY.

To recapitulate briefly, the diagnosis of malignant disease of the mouth, namely carcinoma and sarcoma, is largely dependent upon the characteristic feature of rapid increase in intensity to a fatal ending. The associated manifestations of this increase are inevitable destruction of the normal tissue in the region, with the attendant loss of function and replacement with tumor tissue; toxic poisoning and hemolytic action upon the blood, producing progressive anemia, loss in body weight, and increasing prostration. The regional or general development of similar tumors, together with the more rapid deterioration of the health of the patient, is a further manifestation of the disease, and usually occurs.

The characteristic pain in malignant disease, which is sharp and lancinating and late in development, or absent until inflammatory complications occur, cannot be considered as a diagnostic factor.

The history of frequent occurrences of similar diseases in members of a patient's family, or their entire absence, should not have much influence in determining the diagnosis.

